# Peli1 signaling blockade attenuates congenital zika syndrome

**DOI:** 10.1371/journal.ppat.1008538

**Published:** 2020-06-16

**Authors:** Huanle Luo, Guangyu Li, Binbin Wang, Bing Tian, Junling Gao, Jing Zou, Shuizhen Shi, Shuang Zhu, Bi-Hung Peng, Awadalkareem Adam, Ariza Martinez, Kimberly Hein, Evandro R. Winkelmann, Yoseph Mahmoud, Xiaofei Zhou, Chao Shan, Shannan Rossi, Scott Weaver, Alan D. T. Barrett, Shao-Cong Sun, Wenbo Zhang, Pei-Yong Shi, Ping Wu, Tian Wang

**Affiliations:** 1 Department of Microbiology & Immunology, University of Texas Medical Branch, Galveston, TX, United States of America; 2 Department of Internal Medicine, University of Texas Medical Branch, Galveston, TX, United States of America; 3 Sealy Center for Molecular Medicine, University of Texas Medical Branch, Galveston, TX, United States of America; 4 Department of Neuroscience, Cell Biology and Anatomy, University of Texas Medical Branch, Galveston, TX, United States of America; 5 Department of Biochemistry & Molecular Biology, University of Texas Medical Branch, Galveston, TX, United States of America; 6 Department of Ophthalmology and Visual Sciences, University of Texas Medical Branch, Galveston, TX, United States of America; 7 Department of Immunology, The University of Texas MD Anderson Cancer Center, Houston, Texas, United States of America; 8 Institute for Human Infections & Immunity, University of Texas Medical Branch, Galveston, TX, United States of America; 9 Department of Pathology, University of Texas Medical Branch, Galveston, TX, United States of America; 10 Sealy Institute for Vaccine Sciences, University of Texas Medical Branch, Galveston, Texas, United States of America; Mount Sinai School of Medicine, UNITED STATES

## Abstract

Zika virus (ZIKV) infects pregnant women and causes devastating congenital zika syndrome (CZS). How the virus is vertically transmitted to the fetus and induces neuronal loss remains unclear. We previously reported that Pellino (Peli)1, an E3 ubiquitin ligase, promotes p38MAPK activation in microglia and induction of lethal encephalitis by facilitating the replication of West Nile virus (WNV), a closely related flavivirus. Here, we found that Peli1 expression was induced on ZIKV-infected human monocytic cells, peripheral blood mononuclear cells, human first-trimester placental trophoblasts, and neural stem cell (hNSC)s. Peli1 mediates ZIKV cell attachment, entry and viral translation and its expression is confined to the endoplasmic reticulum. Moreover, Peli1 mediated inflammatory cytokine and chemokine responses and induced cell death in placental trophoblasts and hNSCs. ZIKV-infected pregnant mice lacking Peli1 signaling had reduced placental inflammation and tissue damage, which resulted in attenuated congenital abnormalities. Smaducin-6, a membrane-tethered Smad6-derived peptide, blocked Peli1-mediated NF-κB activation but did not have direct effects on ZIKV infection. Smaducin-6 reduced inflammatory responses and cell death in placental trophoblasts and hNSCs, and diminished placental inflammation and damage, leading to attenuated congenital malformations in mice. Collectively, our results reveal a novel role of Peli1 in flavivirus pathogenesis and suggest that Peli1 promotes ZIKV vertical transmission and neuronal loss by mediating inflammatory cytokine responses and induction of cell death. Our results also identify Smaducin-6 as a potential therapeutic candidate for treatment of CZS.

## Author summary

We previously reported that Pellino (Peli)1, an E3 ubiquitin ligase mediates p38MAPK activation in microglia and induces lethal encephalitis by facilitating replication of a mosquito -borne flavivirus, West Nile virus (WNV). Zika virus (ZIKV), a closely related flavivirus, causes devastating congenital zika syndrome (CZS) in pregnant women. How ZIKV is vertically transmitted to the fetus and induces neuronal loss remains unclear. Here, we found that Peli1 expression was enhanced in human monocytic cells, peripheral blood mononuclear cells, first-trimester placental trophoblasts and neural stem cell (hNSC)s following ZIKV infection. Peli1 expression colocalized with the endoplasmic reticulum and double-stranded RNA in ZIKV-infected cells and was required for ZIKV cell attachment and replication. Peli1 knockdown in placental trophoblasts inhibited ZIKV replication and decreased inflammatory cytokine responses and cell death. ZIKV-infected pregnant mice lacking Peli1 signaling showed reduced placental inflammation and tissue damage, which resulted in attenuated congenital abnormalities. Furthermore, Smaducin-6, a membrane-tethered Smad6-derived peptide, blocked Peli1-mediated NF-κB activation, but not ZIKV replication. Smaducin 6 inhibited Peli1-mediated inflammatory cytokine responses and cell death in placental trophoblasts and hNSCs, and attenuated congenital malformations in mice. Collectively, our results reveal a novel role of Peli1 in flavivirus pathogenesis and suggest that Peli1 promotes ZIKV vertical transmission and neuronal loss by mediating inflammatory cytokine responses and induction of cell death.

## Introduction

Zika virus (ZIKV), a recently emerging mosquito-borne pathogen, belongs to the *Flavivirus* genus and the family of *Flaviviridae*, which include several other important human pathogens, such as dengue virus (DENV), West Nile virus (WNV) and yellow fever virus (YFV). The virus was initially isolated in Uganda in 1947 [1] and later caused outbreaks in Federated States of Micronesia in 2007, and in Pacific islands in 2013 [2,3]. In 2015, ZIKV was confirmed to cause an outbreak in Brazil [4] and it subsequently spread to the Americas and Caribbean with more than 1 million human infections [5]. ZIKV has been linked to the neurological autoimmune disorder Guillain-Barre syndrome in adults and congenital Zika syndrome (CZS) in fetuses and infants, including microcephaly, spontaneous abortion, and intrauterine growth restriction (IUGR) [6,7]. Neither antiviral treatments nor vaccines are currently available for humans.

Several *in vivo* small animal models have been used to recapitulate CZS in humans. ZIKV infection at embryonic day 6.5 (E6.5) or E7.5 in either wild-type (WT) immunocompetent mice treated with MAR1-5A3, a blocking anti-Ifnar monoclonal antibody, or in Ifnar1 deficient mice can recapitulate the CZS phenotype *in vivo* [8]. Pregnant WT C57BL/6 mice and Swiss Jim Lambert (SJL) mice are also susceptible to ZIKV infection, and exhibit fetal abnormalities or severe IUGR [9–11]. Vaginal infection during early pregnancy or direct injection into one side of the cerebroventricular of WT pregnant dams on E13.5 led to infection of fetal brain and induction of microcephaly [12,13]. There are also several *in vitro* models of CZS, including cultured human neural stem or progenitor cells (hNS/PCs), induced pluripotent stem cell-derived neurospheres or brain organoids, and acute slices. Cultured hNS/PCs were more susceptible to ZIKV replication than mature cortical neurons. ZIKV infection in hNPCs increases cell death and dysregulates cell-cycle progression, resulting in attenuated cell growth [14]. ZIKV replication in cortical neurospheres and organoids also induces cell death, and reduces proliferation and the volume of the organoids [10,15]. Furthermore, placental trophoblasts are epithelial cells that invade and remodel the uterine wall during placentation. Both primary and placental trophoblast cell lines have been used to study ZIKV infection in placenta [16,17]. Despite the large gain in knowledge, how the virus is transmitted to the fetus and induces neuronal loss and whether intervention during pregnancy can protect the host from CZS remains largely unknown.

Pellino (Peli)-1, an E3 ubiquitin ligase, is a positive regulator in pathogen recognition receptor (PRR)-mediated inflammatory cytokine responses, either by activation of nuclear factor (NF)-κB or by promoting the degradation of TNF receptor-associated factor 3 (Traf3). Peli1 also modulates necroptosis and apoptosis [18]. It is expressed on many cell types and is enriched in the central nervous system (CNS) tissues [19,20]. We have previously reported that Peli1 promotes mitogen-activated protein kinase (MAPK) activation in microglia primarily by facilitating WNV replication in the CNS [21]. In this study, we investigated the role of Peli1 using *in vivo* and *in vitro* models of CZS. Our results suggest that Peli1 is involved in multiple steps of the ZIKV life cycle. It also mediates inflammatory cytokines response and induction of cell death in ZIKV-infected human placental trophoblasts and hNS/PCs. In particular, Peli1 promotes ZIKV vertical transmission by mediating placental inflammation and tissue damage. Treatment with Smaducin-6, a membrane-tethered Smad6-derived peptide, blocked Peli1-induced NF-κB activation, reduced ZIKV- induced placental inflammation and tissue damage, and ultimately attenuated congenital malformations.

## Results

### Peli1 is associated with ZIKV infection in humans

**Fig 1:**
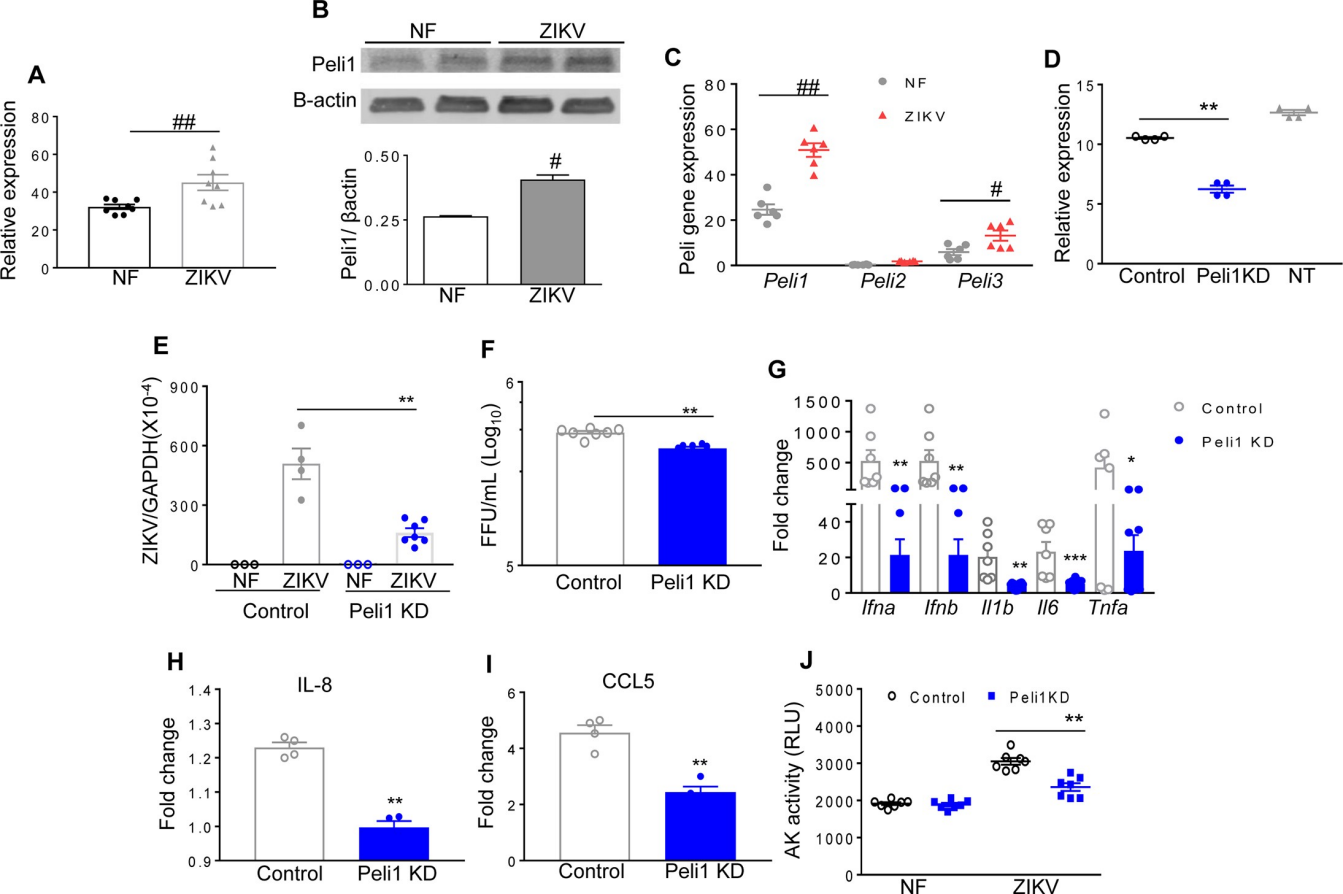
Peli1 expression in human monocytic cells, PBMCs and placental trophoblasts following ZIKV infection. **A.** THP-1 cells were infected with ZIKV-FSS13025 at MOI of 1. RNA levels of Peli1 at day 4 were determined by qPCR assay. Data are presented as means ± SEM, n = 8. **B.** Western blot assays of Peli1 and β-actin expression on PBMCs of a healthy donor infected with ZIKV FSS13025 (MOI = 1) at day 3 pi. Top panel: Western blot image. Bottom panel: Image intensity was quantified using the Odyssey Infrared Imaging system and the ratios of Peli1/ β-actin are presented (n = 2). **C.** HTR8 cells were infected with ZIKV-FSS13025 at MOI of 1. RNA levels of Peli1-3 at day 6 were determined by qPCR assay. Data are presented as means ± SEM, n = 6. ^##^ P < 0.01 or ^#^ P < 0.05 compared to non-infected (NF) group (Unpaired t test). **D-J.** HTR8 cells were treated with control or Peli1 siRNA (Peli1KD) or non-treated (NT), infected with ZIKV-FSS13025 at 24 h and harvested at day 4 post infection (pi). **D.** RNA levels of Peli1 were determined at 24 h post treatment by qPCR assay, n = 4. **E-F.** Viral load was measured at day 4 pi by qPCR and FFA. n = 3–7. **G.** Cytokine production was measured by qPCR at day 4 pi. n = 7–12. **H-I.** Chemokine levels were determined by Bioplex. **J.** Cell death was determined at day 4 pi by the activity of cell-associated adenylate kinase released in cell culture supernatants, n = 7. Data are presented as means ± SEM. ** P < 0.01 or *P < 0.05 compared to control group (Unpaired t test).

We have previously reported that ZIKV replicates in human monocytic cells at a low rate [22]. Here, we noted that Peli1 expression was 40 to 58% higher on human monocytic cells (THP-1 cells) and peripheral blood mononuclear cells (PBMCs) following ZIKV infection (**Fig 1A and 1B**). As ZIKV-induced congenital diseases have been associated with viral infection during the first-trimester of pregnancy [23,24], we next examined Peli1 expression on HTR8 cells, which are human first trimester extravillous trophoblast cells permissive to ZIKV infection [16]. We found that Peli1 expression was significantly enhanced on HTR8 cells at day 6 post infection (pi, **Fig 1C**). The levels of Peli3, another member of the Peli family, were also modestly increased. We next performed siRNA knockdown to reduce Peli1 expression by 41% (**Fig 1D**) in HTR8 cells followed by infection with the clinical Asian-lineage FSS13025 strain (ZIKV-FSS13025). As shown in **Fig 1E and 1F**, knockdown of Peli1 decreased viral loads in ZIKV-infected HTR8 cells compared to control siRNA treated cells as measured by quantitative (q)PCR and focus forming assay (FFA). It also diminished mRNA levels of type I IFNs (*Ifna*, *Ifnb*), and inflammatory cytokines (*Il6*, *Il1b*, and *Tnfa*), and the production of chemokines (IL-8 and CCL5) (**Fig 1G–1I**). ZIKV infection is known to cause cell death [14,25]. Peli1 knockdown in HTR8 cells led to reduced cell death rates following ZIKV infection (**Fig 1J**). Collectively, these results suggest that Peli1 expression is upregulated in ZIKV- infected human PBMCs and monocytic cells, and it promotes ZIKV infection and induction of inflammation and cell death in human placental trophoblasts.

### Peli1 promotes ZIKV infection and placenta inflammation and exacerbates congenital abnormalities in mice

**Fig 2:**
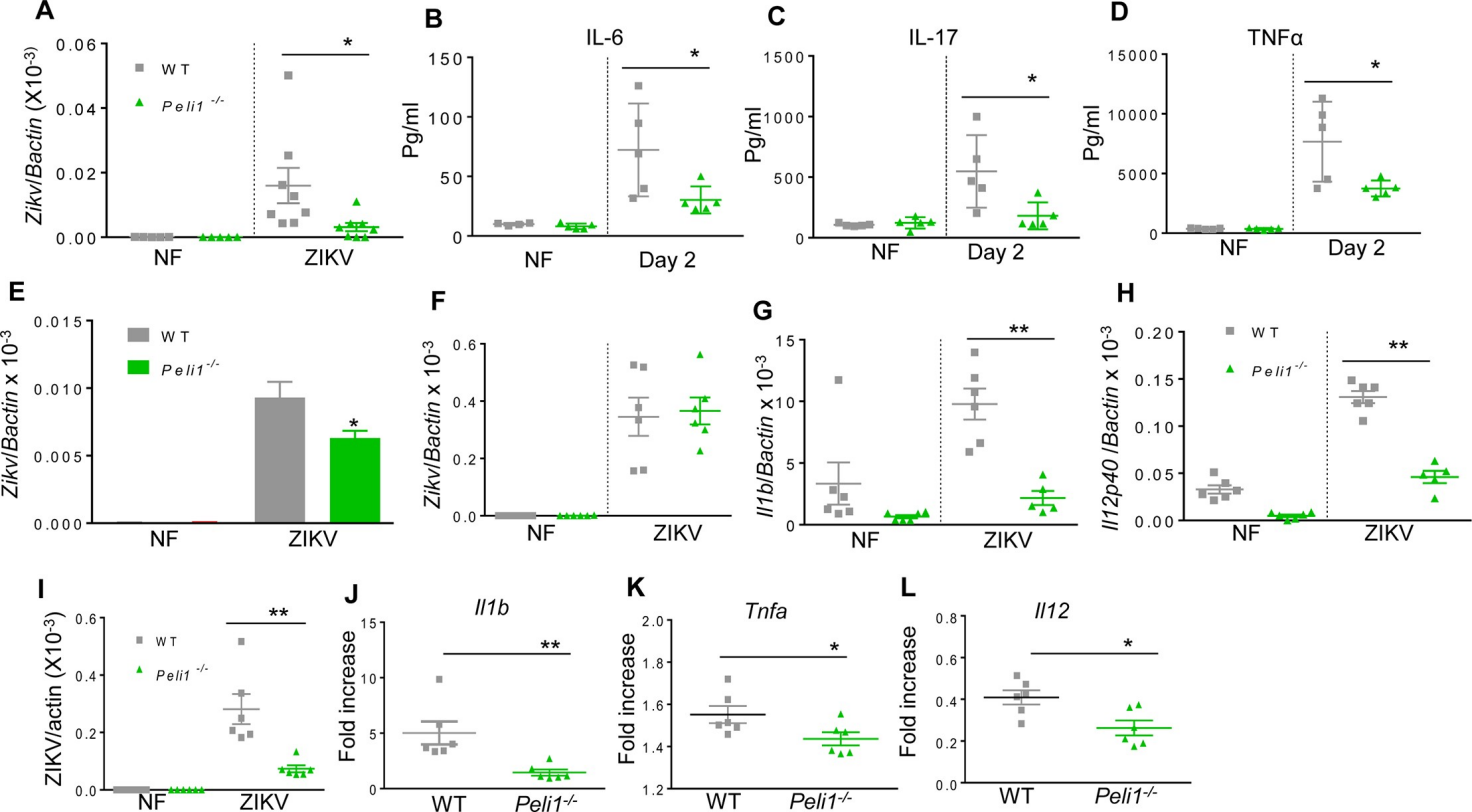
Peli1 promotes ZIKV infection and induction of innate immunity in macrophages. **A- D.** WT and *Peli1*^*-/-*^ mice were infected with ZIKV-FSS13025. **A.** Viral load in the spleen at day 2 pi and non-infected (NF) mice was determined by qPCR assay. Data are presented as mean ± SEM, n = 5–8. **B-D.** Blood cytokine levels at day 2 were determined by Bioplex. Data are presented as the means ± SEM of 4–5 samples from 2 independent experiments. **E-F.** Viral load of ZIKV-FSS13025 -infected macrophages (MOI 0.5) and DCs (MOI 5) was measured by qPCR assays. n = 5–6 per group. **G-H.** Cytokine RNA levels of DCs were measured at indicated time points by qPCR. Data represent means ± SEM of 5–6 samples. **I-L.** WT and *Peli1*^*-/-*^ macrophages were blocked with (MAR1-5A3, 25ug/ ml) followed by ZIKV-FSS13025 infection (MOI = 5). Viral load (**I)** and cytokine RNA levels (**J-L**) were measured at day 4 pi by qPCR. Data are presented as the fold increase compared to mock- infected (means ± SEM), n = 6. ** P < 0.01 or *P < 0.05 compared to WT group (Unpaired t test).

We next investigated the role of Peli1 in ZIKV pathogenesis in the murine model. Adult wild-type (WT) immunocompetent mice are resistant to ZIKV infection [26–28]. We injected 5–6 week old WT and *Peli1*^*−/−*^ mice intraperitoneally (i.p.) with 1 x10^6^ PFU ZIKV-FSS13025. On day 2 pi, a lower viral load was noted in *Peli1*^*−/−*^ mouse spleens compared to WT controls (**Fig 2A**). The blood levels of inflammatory cytokines, including IL-6, IL-17, and TNF-α were markedly reduced in the *Peli1*^*−/−*^ group (**Fig 2B–2D**). Macrophages and dendritic cells (DCs) are known to be permissive to ZIKV infection [29,30], so we next examined viral loads in these cell types. Viral loads in *Peli1*^*-/-*^ macrophages, but not in *Peli1*^*-/-*^ DCs were reduced by 30% compared to the WT group (**Fig 2E and 2F)**. The mRNA levels of cytokines in *Peli1*^*-/-*^ DCs, *including Il1b* and *Il12*, were 65% to 75% lower than that of WT DCs (**Fig 2G and 2H**). Thus, Peli1 deficiency in mice with intact type 1 IFNR signaling displayed a partial reduction in ZIKV replication and inflammatory immune responses.

**Fig 3:**
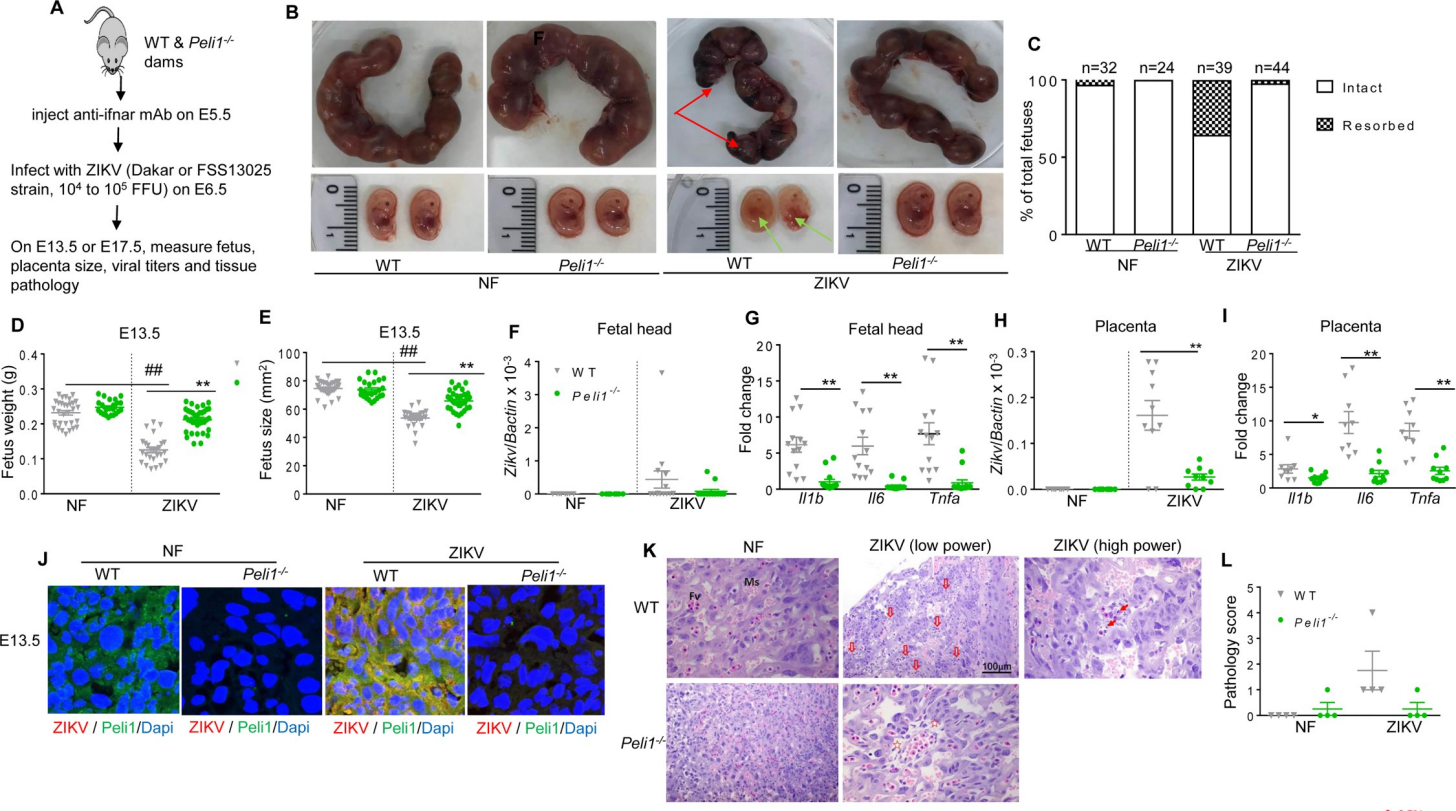
Peli1 mediates inflammatory responses and tissue damage in placenta, and exacerbates birth abnormalities in mice. WT and *Peli1*^*-/-*^ mice were pretreated with MAR1-5A3 at E5.5 followed by infection with 1x 10^4^ FFU ZIKV-Dakar-MA strain one day later. **A.** Schematic representation of experimental procedure. **B.** Representative images of E13.5 uteri (upper panel) and fetuses (lower panel). Partial demise and growth restriction was shown in ZIKV-infected WT dams. Red arrows indicate the left placenta residues. Green arrows show growth restriction of fetuses. **C.** Resorption rates were measured at E13.5. Data are representative of 4–5 independent experiments with one pregnant dam per experiment. **D-E.** The weight (**D**) and size (**E,** CRL x OF diameter) of 24–36 fetuses at E13.5 collected from 4–5 pregnant dams per group. **F-I**. Viral load (**F & H**) and cytokine induction (**G & I**) in fetal heads and placentae collected from 8–14 fetuses per group. **D-I**: ^##^ *P* < 0.01 compared to non-infected (NF) group. ** *P* < 0.01 or *P < 0.05 compared to WT group (Unpaired t test). **J.** Immunodetection of Peli1 (green), and ZIKV antigen (red) on placentae collected at E13.5. Nuclei are counterstained with DAPI (blue). **K-L.** Histology staining of placentae at E13.5. K. Representative images (25X) shown are placentae collected from 4 ZIKV-infected dams per group. Scale bar: 100μm (10X). Damaged vessels indicated by hollow arrows; apoptotic bodies indicated by arrows in higher power image; and mild edema indicated by stars. **L.** Pathology scores were assessed blindly. Data are presented as the means ± SEM.

To increase ZIKV permissiveness, we blocked type I IFNR signaling by anti-ifnar antibody in WT and *Peli1*^*−/−*^ macrophages followed by ZIKV infection. Viral loads in *Peli1*^*−/−*^ macrophages were 75% lower than the WT group (**Fig 2I**). The levels of cytokines, including Il1b, Tnfa, and Il12, were all diminished in *Peli1*^*−/−*^ macrophages following ZIKV infection (**Fig 2J–2L**). We next investigated the role of Peli1 in an *in vivo* model of CZS. WT and *Peli1*^*−/−*^ mice were bred and pretreated with type I IFNR antibody at E5.5 followed by challenge with 1x10^4^ PFU of a mouse-adapted ZIKV strain (ZIKV-Dakar-MA) one day later (**Fig 3A**). On E13.5, partial fetal demise was observed in ZIKV-infected WT group, but barely detected in the *Peli1*^*−/−*^ group. Individual fetuses were also weighed and the size was measured by the crown-rump length and the occipito-frontal (CRL x OF) diameter of the fetal head. Fetal growth restriction was noted in the WT group, but this effect was significantly attenuated in the *Peli1*^*−/−*^ group (**Fig 3B–3E**). Similar fetal demise and growth restriction were observed for fetuses collected on E17.5 after infection with ZIKV-FSS13025 strain (**S1A to S1C Fig)**. Viral loads were barely detected in fetal head of the two groups of mice at either E13.5 (**Fig 3F**) or E17.5 (**S1D Fig**), though there was a decreased viral load in fetal tissues of the *Peli1*^*−/−*^ group following a higher infectious dose (1x10^5^ PFU) of ZIKV-Dakar-MA strain (**S1E Fig**). In contrast, viral loads in placenta were diminished in the *Peli1*^*−/−*^ group (**Fig 3H**). Furthermore, inflammatory cytokine responses in both fetal head and placenta tissues were significantly diminished in the *Peli1*^*−/−*^ group compared to the WT group (**Fig 3G and 3I**). Immunofluorescence staining revealed enhanced Peli1 expression that was co-localized with ZIKV antigens detected in WT placenta tissues on E13.5. Both Peli1 and ZIKV antigens were diminished in the *Peli1*^*−/−*^ group (**Fig 3J**). Histology staining showed that in the placentae of WT mice, many fetal blood vessels were damaged, accompanied with cell death (**Fig 3K**). Compared to the non-infected WT group, fetal red blood cells, large and nucleated, were fewer in the area with injured vessels and some were apoptotic in addition to endothelial injury. However, placentae from the *Peli1*^*−/−*^ mice showed normal to mild vascular edema. Furthermore, no apoptosis or fetal blood vessel damage was observed (**Fig 3K and 3L**). Finally, there were lower mRNA levels of inflammatory cytokines (*Il6* and *Il1b*) detected in *Peli1*^*−/−*^ maternal blood (**S1F and S1G Fig**). Collectively, Peli1 deficiency in mice with a defective type I IFNR signaling contributes to markedly lower viral loads, reduced inflammation and tissue damage in placenta, and attenuated congenital malformation phenotype.

### Peli1 is involved in multiple steps of the ZIKV life cycle

**Fig 4:**
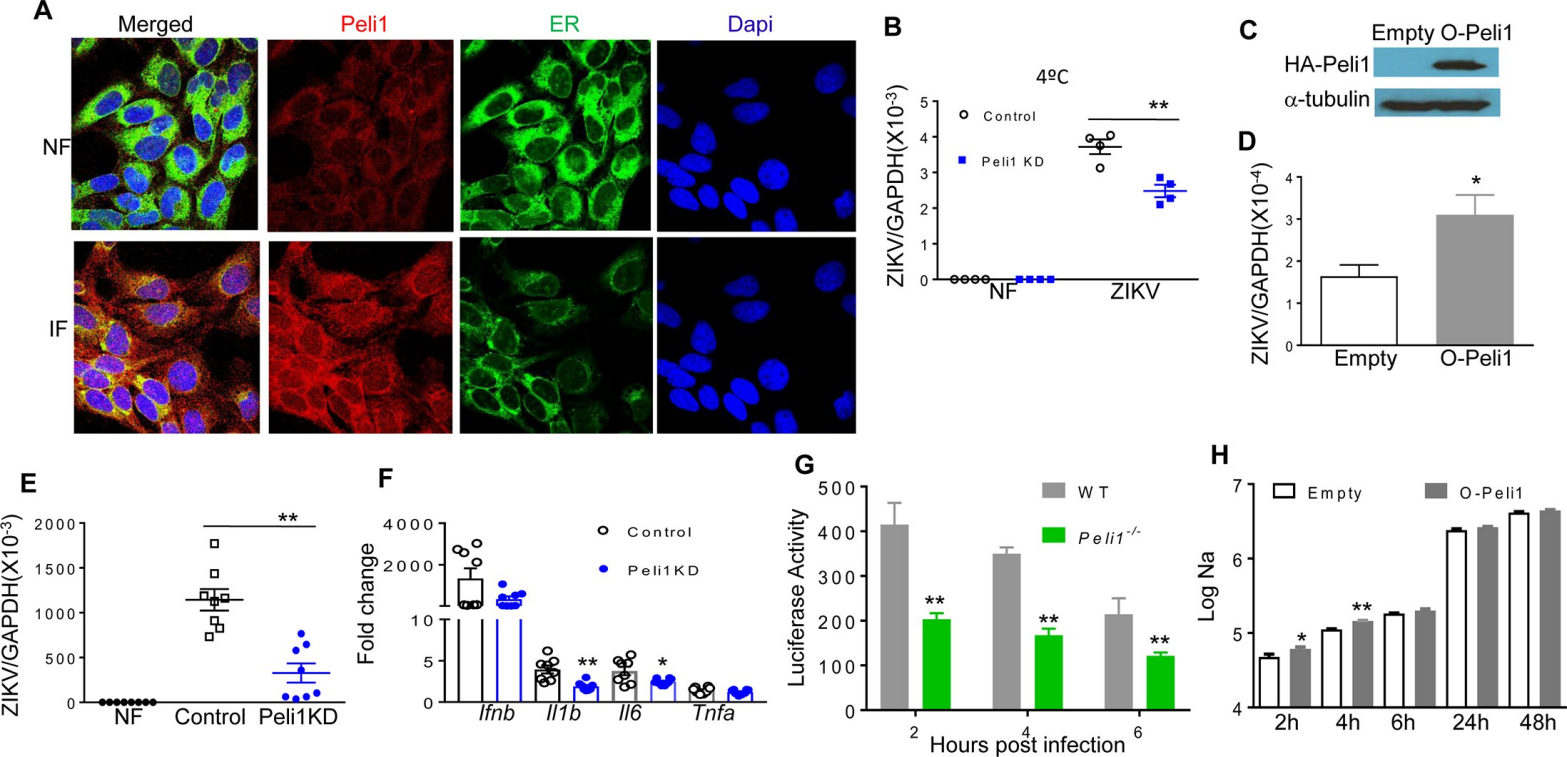
Peli1 is involved in multiple stages of the ZIKV life cycle. **A.** HTR8 cells were infected with ZIKV-FSS13025 (MOI = 10). At 24 h pi, cells were fixed with 4% paraformaldehyde. One representative image of three stained samples was shown. Immunodetection of ER (green), Peli1 (red), and Dapi (blue). **B.** HTR8 cells were treated with control and Peli1 siRNA (Peli1KD) for 24 h, infected with ZIKV-FSS13025 (MOI = 10) for 1 h at 4°C, washed and intracellular viral RNA measured by qPCR in the attachment assay. n = 4. **C.** Western blot analysis of Peli1 and α-tubulin expression on 293T cells overexpressing Peli1 (O-Peli1) or empty vector (empty). **D.** Viral entry assay in 293T cells overexpressing Peli1 (O-Peli1) or an empty vector. Cells were infected with ZIKV-FSS13025 (MOI = 10) for 1 h at 4°C, washed, and were subsequently resuspended in medium and incubated at 37°C for 3 h. Cells were washed to determine intracellular viral RNAs. n = 5. **E-F.** HTR8 cells were infected at MOI of 1 with viruses passaged once in control and Peli1KD treated HTR8 cells. Viral load and cytokine production were measured by qPCR at day 4. Data are presented as means ± SEM, n = 8. **G-H**. WT and *Peli1*^*-/-*^ mouse macrophages or Peli1 overexpression 293T cells and controls were transfected with infectious ZIKV-Nano reporter RNA. At indicated time points post transfection, cells were assayed for Nano luciferase activity. Data are presented as means ± SEM, n = 4. **B, E-F:** ** P < 0.01 or *P < 0.05 compared to control group (Unpaired t test). **D& H:** ** P < 0.01 or *P < 0.05 compared to 293T cell with an empty vector (Unpaired t test). **G:** ** P < 0.01 compared to WT group (Unpaired t test).

Immunofluorescence staining was next performed following ZIKV infection (MOI 10) of HTR8 cells in order to determine whether Peli1 is involved in the ZIKV life cycle. Peli1 expression was enhanced and it was co-localized with ZIKV antigens at 4 h and 24 h pi (**S2A Fig**). During flavivirus infection, viral genome replication and virion assembly occurs on and within the endoplasmic reticulum (ER) [31]. Indeed, at both 4 h and 24 h pi, Peli1 expression co-localized with the ER marker and dsRNA, which indicates Peli1 is confined to the ER and likely associated with virus replication (**Fig 4A, S2B and S2C Fig**). To determine the role of Peli1 in the ZIKV replication life cycle, we next performed virus attachment assays in HTR8 cells. Control and Peli1 siRNA treated HTR8 cells were incubated with ZIKV-FSS13025 at 4°C for 1 h, allowing the virus to attach to the cell surface. After 1 h incubation, cells were washed to remove unattached virus, and the amounts of virus that had attached to the cell surface were measured. As shown in **Fig 4B**, viral RNA in Peli1 siRNA treated HTR8 cells was significantly decreased compared to control siRNA treated cells, suggesting a role of Peli1 in virus attachment. We next determined the effects of Peli1 overexpression on viral entry. We infected 293T cells overexpressing Peli1 or an empty vector with ZIKV (**Fig 4C**, MOI = 10), and after 1 h incubation at 4°C, the cells were washed and further incubated at 37°C to initiate viral entry. The infected cells were stringently washed at 3 h pi to remove free virus as well as cell surface-associated virus, and the intracellular viral RNA was found to be higher in 293T cells overexpressing Peli1 compared to the control group (**Fig 4D**). To determine if Peli1 is involved in other steps of the ZIKV life cycle, HTR8 cells were infected with equal titers of ZIKV passaged once in control or Peli1 siRNA treated HTR8 cells respectively. On day 4, ZIKV infection was decreased in HTR8 cells infected with virus passaged in Peli1 siRNA treated cells (**Fig 4E**). Cytokine production, including IL-1β and IL-6 but not IFN-β and TNFα, was also reduced (**Fig 4F**). Overall, these results confirm that Peli1 also contributes to ZIKV infectivity. To further investigate the role of Peli1 in ZIKV infection, macrophages from WT and *Peli1*^*-/-*^ mice were transfected with infectious ZIKV-Nano reporter RNA, as shown in **Fig 4G.** The viral RNA translation levels in *Peli1*^*-/-*^ macrophages were lower than that in WT macrophages, as reflected by the luciferase signals at 2 h, 4 h, and 6 h, indicating that Peli1 is important for ZIKV viral RNA translation. Using the same method, we measured Peli1 overexpression during ZIKV infection of 293T cells overexpressing Peli1 or empty vector. At 2 h and 4 h post transfection, Peli1 overexpression increased early viral RNA translation, as indicated by the luciferase signals (**Fig 4H**). In summary, these results indicate that Peli1 expression is induced following ZIKV infection and it is involved in the cell attachment, entry, and translation stages of ZIKV life cycle.

### Smaducin-6 treatment decreases inflammatory responses and cell death and attenuates congenital malformations

**Fig 5:**
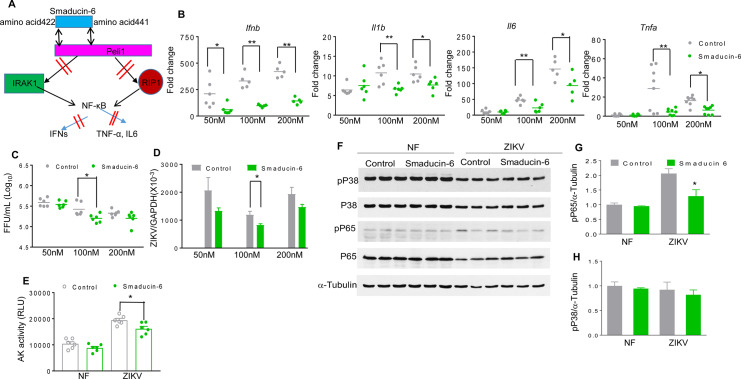
Smaducin-6 treatment in first trimester placental trophoblasts during ZIKV infection. **A.** Schematic representation of Smaducin-6 inhibition of NF-KB activation. **B-H.** HTR8 cells were infected with ZIKV-FSS13025 and treated with Smaducin-6 or control peptides at 1 h pi. **B.** Cytokine levels at day 4. Data are presented as fold increase compared to mock- infected and represent the means ± SEM of 5–8 samples. **C-D.** Viral load was measured at day 4 by FFA (**C**) and qPCR (**D**). Data represent the means ± SEM of 6–11 samples. **E.** Cell death was determined at day 4 by the activity of adenylate kinase in culture supernatants. Data are presented as means ± SEM, n = 6. **F-H.** Western blot assay for p38MAPK and NF-κB activation in HTR8 cells at day 3 pi. **F.** One representative experiment was shown, n = 3. **G-H.** Densitometric analysis of western blot data. Data are presented as the ratio of pP65 or pP38 to α-tubulin. ** P < 0.01 or *P < 0.05 compared to control group (Unpaired t test).

Smaducin-6, a membrane-tethered Smad6-derived peptide, was previously reported to block Peli1 function by binding to IRAK1-, RIP1-, and IKKε-mediated TRIF signaling complexes [32] (**Fig 5A**). To investigate whether Smaducin-6 is able to prevent ZIKV-induced IUGR, we first treated HTR8 cells with various concentrations of Smaducin-6 and control peptides 1 h after ZIKV-FSS13025 infection. As shown in **Fig 5B**, Smaducin-6 treatment reduced IFNβ, IL-1β, IL-6, and TNF-α production at various doses in HTR8 cells. Smaducin-6 only modestly decreased viral loads at 100 nM compared to the control group (**Fig 5C and 5D**), which was also accompanied by lower cellular death rates as measured by the activity of cell-associated adenylate kinase released in cell culture supernatants (**Fig 5E and S3A Fig**). Smaducin-6 treatment reduced the phosphorylation levels of the p65 subunit of NF-κB, but did not affect p38 MAPK phosphorylation in ZIKV-infected HTR8 cells (**Fig 5F–5H**). Similarly, we observed inhibitory effects of Smaducin-6 treatment on virus replication and induction of inflammatory responses in HTR8 cells upon infection with the epidemic strain ZIKV-PRV (**S3B and S3C Fig**). To determine the effects of Smaducin-6 on ZIKV life cycle, we performed ZIKV attachment and entry assays in HTR8 cells following treatment with peptide control or Smaducin-6 (100 nM), and infection with ZIKV-FSS13025 (MOI of 10). After 1 h incubation at 4°C, the cells were washed to remove unattached virus, and the amounts of viruses that had attached to the cell surface were measured by qPCR. Viral RNA levels were found to be similar between the treated cells and the control groups (**S4A Fig**). Next, cells were further incubated at 37°C to initiate viral entry. The infected cells were stringently washed at 4 h pi to remove free virus as well as cell surface-associated virus, and the intracellular viral RNA was quantitated. These levels were also found to be at similar levels between Smaducin-6- and control peptide -treated cells (**S4B Fig**). Finally, we inoculated HTR8 cells with equal titers of virus passaged once in ZIKV-infected control or Smaducin-6 treated HTR8 cells respectively. Neither cytokine production nor viral loads were found to be significantly different at day 4 pi (**S4C and S4D Fig**). Combined together, these results indicate that while Smaducin-6 inhibits inflammatory responses upon ZIKV infection, it does not appear to block ZIKV cell attachment, entry, or virus replication.

**Fig 6:**
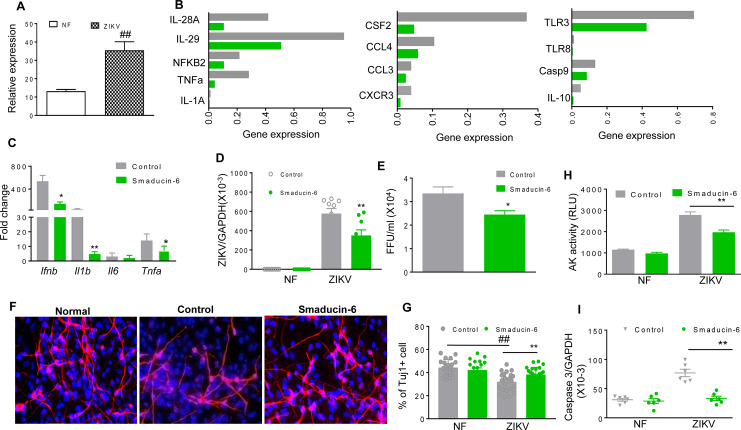
Smaducin-6 treatment in ZIKV-infected hNS/PCs. **A.** hNS/PCs were infected with ZIKV-FSS13025 at MOI of 10. RNA levels of Peli1 at day 4 were determined by qPCR assay. Data are presented as means ± SEM, n = 9. **B-I.** hNS/PCs were infected at MOI of 10 with ZIKV-FSS13025 and treated with 100 nM of Smaducin-6 or control peptides at 1 h pi. **B.** PrimePCR array was performed using day 4 samples. Data show the normalized expression of target genes of control vs. Smaducin-6 treated hNS/PCs. **C.** Cytokine levels were measured at day 4 pi by qPCR. Data are presented as fold increase compared to mock-infected and represent the means ± SEM of three independent experiments, n = 3–9. **D-E.** Viral load was measured at day 4 by qPCR (**d,** n = 9) and FFA (**e,** n = 3, one representative of three independent experiments was shown). **F-G.** Immunodetection of TuJ1 (red) and Dapi (blue). **F.** One representative image. **G.** The number of TuJ1-labeled neurons was blindly counted by NIS-Elements imaging software and the neurons labeled with TuJ1 from 20 randomly selected fields were included for quantitative assessments, n = 21–25. **H.** Cell death was measured by the activity of cell-associated adenylate kinase released in day 4 cell culture supernatants, n = 6. **I.** Caspase 3 levels were measured by qPCR at day 4, n = 6. ^##^ P < 0.01 compared to non-infected (NF) (Unpaired t test). ** P < 0.01 or *P < 0.05 compared to control group (Unpaired t test).

One of the *in vitro* CZS models which support ZIKV replication are hNS/PCs so we examined Peli1 expression on this additional cell type. Peli1 expression was upregulated following ZIKV infection in hNS/PCs derived from human fetal brain tissues (**Fig 6A**). PrimePCR array analysis of hNS/PCs showed that the mRNA levels of inflammatory cytokine related genes (IL-1A, TNFα, IL28A, IL29, NF-KB2), chemokines (CSF2, CCL3, CCL4, CXCR3),TLRs (TLR3, TLR8), IL-10, and Casp9 were all reduced in the Smaducin-6 treated group following ZIKV-FSS13025 infection (**Fig 6B**). Additionally, qPCR assay confirmed the reduction of mRNA levels of *Ifnb*, *Il1b*, and *Tnfa* in Smaducin-6 treated hNS/PCs following ZIKV infection (**Fig 6C**). Viral loads were also reduced following the treatment as measured by qPCR and FFA (**Fig 6D and 6E**). Furthermore, the number of type III β-tubulin-labeled neurons were reduced in the control peptide treated group following ZIKV infection. In comparison, the number of tubulin- positive cells was not affected by ZIKV infection in Smaducin-6 treated group (**Fig 6F and 6G**), which suggests a protective role of Smaducin-6 on cell viability. Indeed, apoptosis rates were decreased following Smaducin-6 treatment as measured by the levels of adenylate kinase (**Fig 6H**) and caspase 3 gene expression in ZIKV-infected cells (**Fig 6I)**. In summary, Smaducin-6 decreases inflammatory cytokine responses, viral load, and cell death in hNS/PCs.

**Fig 7:**
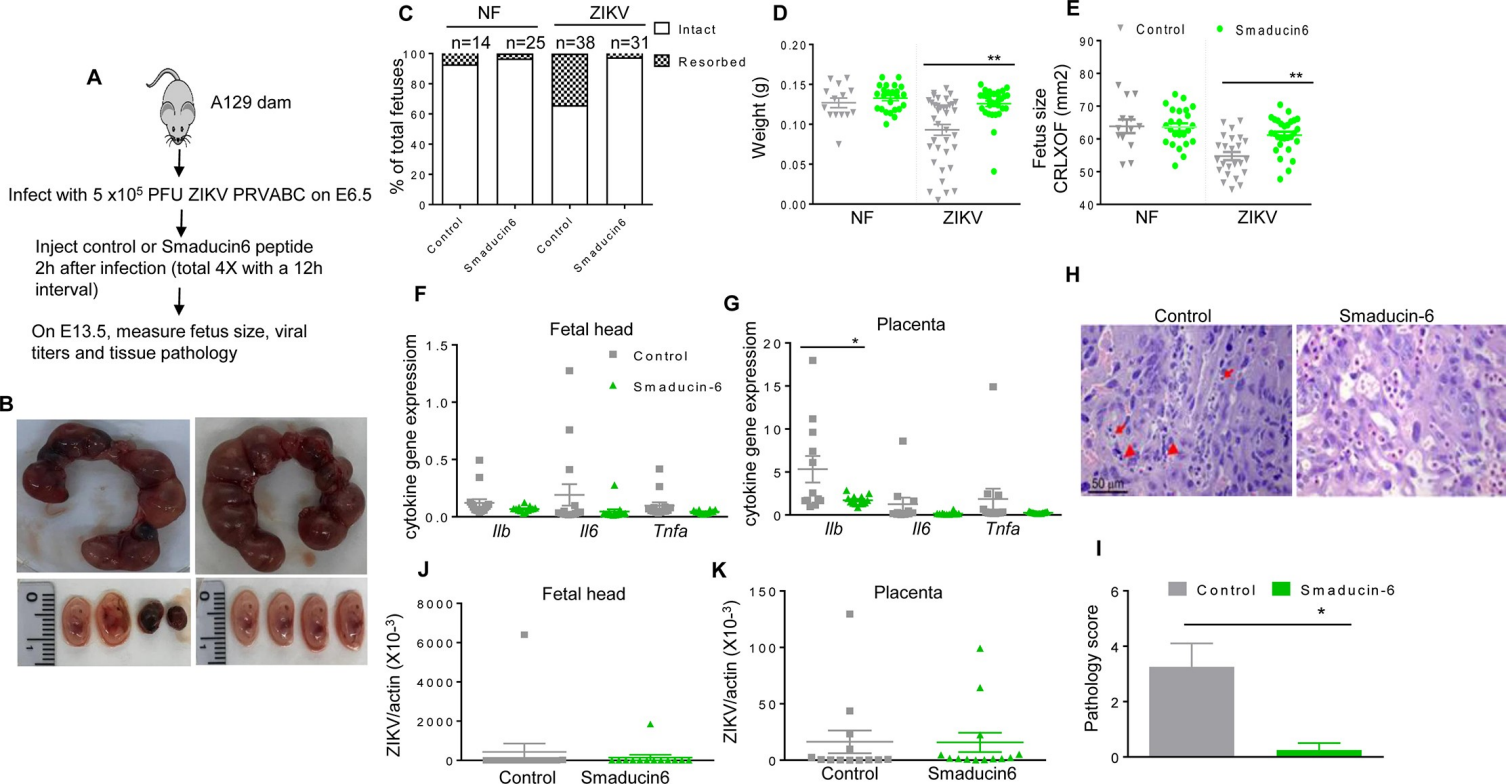
Smaducin-6 treatment attenuates congenital diseases in ZIKV-infected pregnant dams. A129 mice were infected with 5 x10^5^ FFU ZIKV-PRV or PBS on E6.5, followed by injection with control or Smaducin-6 peptide 2 h pi and three additional treatments every 12 h. **A.** Schematic representation of experimental procedure. **B.** Representative images of E13.5 uteri (upper panel) and fetuses (lower panel). **C.** Resorption rates were measured at E13.5. Data are representative of 3–5 pregnant dams. **D-E.** The weight (**D**) and size (**E,** CRL x OF diameter) of 14–38 fetuses at E13.5 collected from 3–5 pregnant dams. **F-K.** Fetal heads and placentae harvested on E13.5. **F-G.** Cytokine production in fetal heads and placentae of 12–15 fetuses pooled from 4–5 experiments. **H-I.** Histology staining of placentae at E13.5. Apoptotic bodies (arrows) and eosinophilic shrunken cells (arrowheads) indicated in control placentae. **H**. Representative images (20X) shown are placentae collected from 4 ZIKV-infected dams per group. Scale bar: 50μm. **I.** Pathology score assessed blindly. **J- K.** qPCR assay of viral loads of 12–15 fetuses per group. For panels **D, E, G, & I:** ** P < 0.01 or *P < 0.05 compared to control group (Unpaired t test).

We next studied the therapeutic effects of Smaducin-6 in mice following ZIKV infection using type I IFNR deficient mice on either C57BL/6 (AB6) or 129 (A129) backgrounds. At day 3 pi, there was a trend of lower viral loads in both spleen and blood of Smaducin-6 treated A129 mice (**S5A and S5B Fig**). Smaducin-6 treated macrophages isolated from AB6 mice showed decreased levels of IL-12, and a trend of reduction on viral loads, but did not affect TNF-α production when compared to the control group (**S5C–S5E Fig**). Interestingly, Smaducin-6 treatment in the *Peli1*^*−/−*^ macrophages blocked with type I IFNR signaling did not affect IL-12 or viral load compared to the control peptide treated *Peli1*^*−/−*^ macrophages (**S5F and S5G Fig**). Thus, Smaducin-6 may inhibit ZIKV infection and induction of innate cytokine responses via interacting with Peli1. Furthermore, timed pregnant A129 mice were inoculated with 5 x 10^5^ PFU of ZIKV-PRV strain on E6.5, and 2 h after the infection, pregnant dams were treated with control peptide or Smaducin-6 as described previously [32] (**Fig 7A**). At E13.5, gross morphological observation of the uteruses showed more fetus demise in control peptide-treated dams compared with Smaducin-6 treated dams (**Fig 7B**). Additionally, 31.6% of the fetuses were absorbed in the control dams, while 0.032% of embryos underwent demise in the Smaducin-6 treated group (**Fig 7C**). In terms of ZIKV-induced IUGR, weight loss and size reduction of fetuses from the control peptide treated group were significantly attenuated in the Smaducin-6 treated group (**Fig 7D and 7E**). Though not statistically significant, we observed a trend of decreased levels of inflammatory cytokines in fetal heads and placentae (**Fig 7F and 7G**). Histology staining also shows that in placentae of control peptide treated mice, many fetal blood vessels were damaged with cell death indicated by apoptotic bodies and eosinophilic shrunken cells (**Fig 7H and 7I**). However, placentae from the Smaducin-6 treated mice showed normal to mild vascular edema. Viral RNA levels in maternal blood and spleen (**S5H Fig**), fetal heads and placentae were found at similar levels in control peptide and Smaducin-6 treated groups (**Fig 7J and 7K**). In summary, these results suggest that Smaducin-6 treatment decreases inflammatory responses, reduces tissue damage in placenta, and attenuates CZS in mice.

## Discussion

The pathogenesis of CZS is currently not clearly understood. In this study, we provided evidence to support the following major findings: First, Peli1 is involved in multiple steps of the ZIKV replication life cycle and its expression is confined to the ER. Second, Peli1-mediated placenta inflammation and tissue damage promote ZIKV vertical transmission, leading to severe birth defects in pregnant mice. Third, Smaducin-6, which blocks Peli1-mediated NF-κB activation, inhibits inflammatory responses and cell death in human placental trophoblasts and hNSCs, reduces placenta inflammation, and attenuates congenital malformations in mice.

ZIKV pathogenesis is known to be associated with its broad tissue and cell tropisms [23,24,33]. Results from human and animal studies suggest ZIKV infection in both maternal and fetal tissues, including cord blood, placenta, and developing fetal and neonatal brains [34–38]. ZIKV also replicates differentially in a wide range of cell types, including decidual fibroblasts and macrophages, trophoblasts, Hofbauer cells, and hNS/PCs [14,15,39–41]. Our data showed that Peli1 expression was enhanced on human PBMCs and human monocyte cells following ZIKV infection. Peli1 is also upregulated on ZIKV-infected first trimester placental trophoblasts and hNS/PCs, which together indicates a role of Peli1 in ZIKV infection in humans.

Inflammation has been recognized as a critical contributor to both normal development and injury in the immature brain. Preterm newborns exposed to intrauterine inflammation are at an increased risk of neurodevelopmental disorders. Induction of pro-inflammatory cytokine and type I IFN responses in placenta trophoblasts leads to cell death [42]. Increasing evidence suggests the association of inflammation with CZS. For example, ZIKV infection in placenta and glial cells causes placenta insufficiency and inflammation, which then induces non-autonomous effects on hNS/PCs and impairs neurogenesis [17,30,43]. ZIKV-induced inflammatory immune responses in human cerebral organoids were accompanied either by the depletion of hNS/PCs, or alterations in neuronal differentiation among human NS/PCs [44]. Elevated levels of placental type I IFNs and IL-1β were reported to promote fetal demise upon congenital ZIKV infection [45,46]. Despite the evidence of inflammation in induction of CZS, the underlying immune mechanisms are not clearly understood. Here, in our murine model, ZIKV infection caused an acute increase of placental Peli1 expression, which was accompanied by placental inflammation, tissue damage, and severe congenital malformations. Peli1 is known to modulate necroptosis and apoptosis [18]. We have also demonstrated that Peli1 mediates type I IFN and IL-1β expression in human placenta trophoblasts and hNS/PCs and promotes cell death. Thus, Peli1 may directly induce cell death or indirectly by mediating the inflammatory cytokine responses in trophoblasts and hNSCs upon ZIKV infection. Furthermore, it was reported that increased levels of placental type I IFNs cause abnormal architecture of the maternal-fetal barrier [45,46]. We also noted that fetal tissues collected from *Peli1*^*-/-*^ dams on E13.5 showed lower viral loads following a high dose of ZIKV infection at E6.5. Combined together, our results suggest that Peli1-mediated placental inflammation and cell death contribute to placental tissue damage, which is likely to promote ZIKV vertical transmission to the fetus.

Smaducin-6 is a membrane-tethered palmitic acid-conjugated peptide composed of amino acids 422–441 of Smad-6. It was reported to interact with Peli1 and disrupt the formation of IRAK1, RIP-1, and IKKε, but not p38MAPK-mediated signaling complexes [32]. Systemic administration of Smaducin-6 in mice inhibited pro-inflammatory cytokine production and apoptosis in cecal-ligation-puncture-induced sepsis, and lipopolysaccharide-induced endotoxemia [32]. In our studies, we found that Smaducin-6 reduces inflammation, and cell death in human placental trophoblasts and in hNS/PCs. It also decreases cell death in the latter two cell types. Furthermore, it diminishes ZIKV-induced inflammation and tissue damage in placenta and attenuates congenital diseases in mice. Although we observed a mild decrease on viral load following the treatment, Smaducin-6 did not block ZIKV cell attachment or viral entry. In addition, it did not have any effects on ZIKV infectivity. The anti-viral activity of Smaducin-6 may block ZIKV translation or, more likely, the outcome of its inhibitory effects on the inflammatory cytokine responses and/or cell death. Importantly, we noted that the effects of Smaducin-6 on ZIKV-infected *Peli1*^*−/−*^ macrophages were diminished. These results indicate the inhibitory effects of Smaducin-6 are mediated via Peli1. We previously reported that Smaducin-6 treatment did not block inflammatory cytokine responses in WNV-infected cells [21], as the virus induced Peli1-mediated inflammatory cytokine responses via activation of p38MAPK. In addition, Peli1 promotes microglia activation and leads to lethal encephalitis primarily via facilitating WNV replication in the CNS [21]. Here, Smaducin-6 treatment was shown to block Peli1-mediated NF-KB activation, but not p38MAPK in ZIKV-infected placental trophoblasts. Thus, Peli1-mediated differential innate immune signaling contributes to various disease outcomes during flavivirus infection.

Several host factors have been linked to ZIKV cell entry. For example, AXL, a member of the TAM receptor family, has been shown to be an attachment factor for ZIKV infection in 293 T cells, keratinocytes, and endothelial cells [47,48]. However, studies in AXL deficient mice suggest that it is not required for ZIKV infection in these tissues [35,37]. TIM1, a glycoprotein that is expressed on endothelial cells, trophoblasts, and Hofbauer cells, was reported to interact with phosphatidylserine and is considered to be another candidate for the ZIKV receptor [17]. We have previously reported that Peli1 is involved in WNV attachment, entry, and virus assembly [21]. Similar to WNV infection, we demonstrate that Peli1 is involved in the initial ZIKV attachment to cells, and viral entry. The ER plays an important role in virus replication, assembly and viral particle release during flavivirus infection [31,49]. We further co-localized Peli1 expression to the ER and its association with the replication activities in ZIKV-infected cells. The re-infection studies indicate Peli1 is involved in other steps of viral life cycle. The transfection studies in *Peli1*^*-/-*^ macrophages or Peli1 over-expressing 293T cells with the infectious nano-luciferase reporter ZIKV suggest Peli1 is also involved in viral translation. Based on these findings, we conclude that Peli1 is involved in multiple steps of the ZIKV life cycle. The underlying mechanism are still under investigation. The ubiquitination is likely to be involved in these processes. Many viruses, including Flaviviruses have evolved strategies to utilize host ubiquitination processes in regulation of their own propagation in the host [50–53]. Several E3 ligases have been reported to mediate virus entry or internalization. For example, Greene W et al showed that E3 ligase c-Cbl mediates the ubiquitination of Kaposi's sarcoma-associated herpesvirus and its cognate receptor integrin β1 and facilitates viral entry [54]. It is also possible that Peli1 interacts with other cellular host factors to facilitate virus internalization. During hepatitis C virus infection, the ubiquitin ligase, Casitas B-lineage lymphoma proto-oncogene B forms a complex with another cellular factor, calpain-5 and the hepatitis C virus entry factor CD81 to support the virus entry into liver cells [55]. Overall, our findings suggest Peli1 contributes to CZS by facilitating ZIKV infection, and promoting ZIKV vertical transmission and neuronal loss. The results from this study have provided important insights into ZIKV pathogenesis, and identified Smaducin-6 as a potential therapeutic candidate for treatment of CZS.

## Materials and methods

### Viruses

The Asian lineage ZIKV FSS13025 strain (ZIKV-FSS13025) [56], ZIKV PRVABC59 (ZIKV-PRV) strain, and ZIKV Dakar strain 41525 (ZIKV-Dakar) were obtained from the World Reference Center for Emerging Viruses and Arboviruses (WRCEVA) at the University of Texas Medical Branch (UTMB) and were amplified once or twice in Vero cells. For ZIKV-Dakar-MA, a NS4B G18R mutation was introduced into ZIKV-Dakar infectious clone based on a previous study [57].

### Mice

C57BL/6 (B6) mice and B6 mice deficient in the IFN-α/β receptor (AB6) were purchased from the Jackson Laboratory. *Peli1*^*−/−*^ mice (on a B6 background) [20] and 129/Sv mice deficient in IFN-α/β receptors (A129) were bred at UTMB. Mice were set up for timed-mating and were intraperitoneally (i.p.) inoculated with 1x 10^4^ to 1 x10^6^ PFU of ZIKV-Dakar-MA, ZIKV-FSS13025, or ZIKV-PRV strain at embryonic day (E) 6.5. In some experiments, mice were injected i.p. with 2 mg of anti-ifnar antibody (MAR1-5A3, Leinco Technologies) at E5.5. Infected mice were monitored twice daily for signs of morbidity. On E13.5 or E17.5, mice were euthanized. Fetus weight and size were measured. Placentae and fetuses were harvested for histopathology and viral load studies.

### Ethics statement

Animal studies were carried out in strict accordance with the recommendations in the Guide for the Care and Use of Laboratory Animals of the National Institutes of Health. All animal experiments were approved by the Animal Care and Use Committee UTMB (Permits #1412070).

### Cells

Bone marrow (BM)-derived macrophages and DCs were isolated as described previously [58]. THP-1 cells, a kind gift from Dr. J. McBride (UTMB, Galveston, TX) were cultured as described in our previous study [22]. PBMCs (Fisher Scientific) and THP-1 cells were infected with ZIKV-FSS13025 at MOI of 1. The trophoblast (HTR-8/SVneo) cell line was purchased from the American Type Culture Collection. Cells were maintained in RPMI 1640 supplemented with 10% heat-inactivated FBS. BM-DCs or macrophages were infected with ZIKV at a MOI of 0.1 to 5, and HTR8 cells were infected at MOI of 1 to 10. The K048 line of human neural stem cells (hNSCs), originally derived from the cortex of an eight-week human fetus, was propagated *in vitro* according to what we described previously [59,60]. Dissociated hNS/PCs were infected for 1 h in suspension with ZIKV (MOI = 10) and were re-plated into T25 flasks. In some experiments, MAR1-5A3 (25 μg/ml, Leinco Technologies) were added 30 min prior to ZIKV infection. Smaducin-6 or Pal-Scram peptides [32] were purchased from Sigma-Aldrich and were used at 50 to 200 nm 1 h after infection. Supernatants and cells were harvested at indicated time points post infection for analysis of viral loads, cell death, and cytokine production. To generate Peli1 overexpressing cells, HEK293T cells were transfected with pMIGR1-IRES-EGFP (empty) or pMGIR1-HA-Peli1-IRES-EGFP(HA-Peli1) along with the packing vector pCL-Ampho. HEK293T cells were infected with the recombinant retroviruses. After 48 h, the transduced cells were enriched by flow cytometry cell sorting on the basis of EGFP expression.

### Quantitative PCR (qPCR)

Samples were re-suspended in Trizol (Invitrogen) for RNA extraction. cDNA was synthesized by using a qScript cDNA synthesis kit (Bio-Rad). The sequences of the primer sets for ZIKV, Peli1, Peli2, Peli3, and cytokines cDNA and PCR reaction conditions were described previously [20,61,62]. The assay was performed in the CFX96 real-time PCR system (Bio-Rad). Gene expression was calculated based on C_t_ values by using the formula 2^ ^-[C^t^(target gene)-C^t^(GAPDH or β-actin)]^ [63].

### Western blot

Protein of ZIKV-infected HTR8 cells or PBMCs was dissolved in 1X SDS loading buffer, separated on 10% SDS-PAGE gels, electroblotted onto PVDF membranes, probed with primary and secondary antibodies, and detected using the enhanced chemiluminescence (ECL) system (Pierce). The following primary antibodies were used: phospho-p38 (Thr180/Tyr182, #9211s), p38 (#9212), Phospho-NF-κB RelA (Ser 468, #3039) and NF-κB (#3034) were all purchased from Cell Signaling Technology (Beverly), anti-Pellino1 (Santa Cruz Biotechnology, sc-271065), anti-α-Tubulin (Sigma-Aldrich), and anti-β-actin (Abcam). In some experiments, the blots were incubated with IRDye 700-labeled anti-mouse IgG Ab or IRDye 800-labeled anti-rabbit IgG Ab (LICOR Biosciences, Lincoln, NE). The immune complexes were then quantified using the Odyssey Infrared Imaging system (LICOR Biosciences, Lincoln, NE).

### Focus-forming assay (FFA) for viral titer

Vero cells were incubated with sample dilutions for 1 h. A semi-solid overlay containing 0.8% methylcellulose (Sigma-Aldrich), 3% fetal bovine serum, 1% Penicillin-Streptomycin, and 1% L-glutamine was added. At 48 h, the overlay was removed. Cells were washed with PBS, fixed with 1:1 of acetone: methanol solution for at least 30 min at -20°C and were subjected to immunohistochemical (IHC) staining with either a ZIKV hyperimmune mouse ascitic fluid (WRCEVA) or 4G2 antibody followed by goat anti-rabbit HRP-conjugated IgG (KPL) at room temperature for 1 h. After addition of secondary antibody, cells were incubated with a peroxidase substrate (Vector Laboratories) until color developed. The number of foci was determined and used to calculate viral titers expressed as FFU/ml.

### ZIKV attachment and entry assay

HTR8 cells were infected with ZIKV-FSS13025 (MOI = 10) at 4°C for 1 h, treated with Smaducin-6 or control peptide (100 nM) for 1 h, allowing the virus to attach to the cell surface. Infected cells were next washed three times with cold PBS to remove unbound virions. Cell surface-associated viruses were removed by washing with cold alkaline-high-salt solution (1 M NaCl and 50 mM sodium bicarbonate, pH 9.5). After two washes with cold PBS, the cells were harvested, and suspended in 3 ml RPMI medium containing 2% FBS. Total cells were collected by centrifugation at 1,000 × g for 5 min. The cell pellets were resuspended in Trizol for RNA extraction to measure viral titer by qPCR. For the entry assay, after washing the unbound virus, fresh medium containing Smaducin-6 or control peptide (100 nM) were added, and incubated at 37°C for 4 h. The infected cells were stringently washed to remove free virus as well as cell surface-associated virus, and the intracellular viral RNA was quantified by qPCR[64].

### RNA transfection

293T cells and WT or *Peli1*^*−/−*^ BM-macrophages were seeded in 24-well plates. On the next day, cells were transfected with 1 μg per well of infectious Nano-luciferase reporter ZIKV [64] by using TransIT-mRNA transfection reagent (Mirus Bio LLC) according to the manufacturer’s instructions. At 2 h, 4 h, and 6 h post transfection, the cells were assayed for Nano luciferase activity using a Nano-Glo Luciferase Assay (Promega).

### Immunofluorescence staining

For Tuj1staining, on day 2 pi, hNS/PCs were seeded into 24-well plates pre-coated with 0.01% poly-D-lysine and 1μg/cm^2^ laminin (Invitrogen). Cells were cultured in ELL media (20 ng/mL EGF, 10 ng/mL LIF, and 1 μg/mL laminin) for 4 days, and switched to DMEM/F12 medium with B27 for 9 days with half of the medium replaced every 3 to 4 days. Differentiated hNS/PCs were fixed with 4% paraformaldehyde, and stained with mouse monoclonal anti-neuronal class III β-tubulin (Tuj1, Covance, 1:2000) followed by goat anti-mouse IgG conjugated with Alexa Fluor 568 (Invitrogen, 1:400). Nuclei were counterstained with Dapi (Sigma, 1:2000). Images were acquired with a Nikon 80i epifluorescent microscope. The number of TuJ1^+^ neurons from 20 randomly selected fields was blindly counted using the NIS-Elements imaging software. For HTR8 cells staining, cells were re-plated and grown on glass cover slips pretreated with rat tail collagen (Roche Applied Sciences) in a 6-well culture dish. HTR8 cells were infected with ZIKV (MOI 1 to 10). At the indicated time points pi, cells were fixed with 4% paraformaldehyde, incubated with 0.1 M ammonium chloride for 10 min, and permeabilized using 0.5% Triton-100. Cells were blocked in working buffer (5% goat serum, 0.1% IGEPAL CA-630, 0.05% NaN3, and 1% BSA) and incubated with primary antibodies, including rabbit anti ZIKV NS3 (GTX133320, GeneTex, Inc), 4G2 (UTMB WRCEVA), rabbit anti-Calreticulin antibody (ab2907,Abcam), mouse anti-Peli1 (sc-271065, Santa Cruz biotechnology), rabbit Peli1 antibody (provided by Dr. SC Sun, UT MD Anderson), and anti-dsRNA IgG2a monoclonal antibody (J2-1820, Scicons) in a 1:500 dilution for overnight at 4°C. HTR8 cells were washed and stained with Alexa Fluor 488- or 568-conjugated goat anti-rabbit IgG (5 μg/mL, Life Technologies) for 1 h. Nuclei were counterstained with Dapi for 20 min and the mounted slides were visualized with a LSM880 fluorescence confocal microscope (63X). For placenta sections staining, antigen retrieval of formalin-fixed paraffin-embedded sections was performed in Tris-EDTA buffer. Placenta sections were next blocked using working solution of M.O.M. mouse Ig blocking reagent (Vector Laboratories) followed by incubation with primary antibodies including mouse ZIKV NS3 (GeneTex, Inc), and rabbit anti-Peli1 antibody in working solution of M.O.M. diluent (1:500, Vector Laboratories) for 1 h at room temperature. Normal anti-rabbit IgG were used as controls. After washing, placenta sections were stained with Alexa Fluor 488- or 568-conjugated goat anti-rabbit IgG (5 μg/mL, Life Technologies) in working solution of M.O.M. diluent for 1 h at room temperature. Nuclei were counterstained with Dapi and mounted slides visualized with a LSM880 fluorescence confocal microscope (20X).

### Cytokine Bio-Plex

Tissue culture supernatants or mouse sera were collected for analysis of cytokine production by using 5-plex Bio-Plex Pro Human Cytokine Assays (Bio-Rad) and Th17 6-Plex Pro mouse cytokine assay (Bio-Rad), respectively.

### PrimePCR array

A 45-gene PrimePCR assay (Biorad) was performed following the manufacturer’s protocol. Briefly, RNA was purified from non-infected and viral-infected cells using an RNeasy extraction kit (Qiagen) and quantitated by spectrometry. cDNA was synthesized by using iScript family reverse transcription kits and then loaded onto 96-well PCR array plates for amplification on the CFX96 real-time PCR system (Biorad). A total of 45 genes were assayed as described previously [16]. Data analysis was performed by using CFX manager software. Four genes, specifically SOCS1, NFKB1, TGFB1, and GAPDH, were selected as reference genes for PrimePCR array.

### Histology

Placentas removed from E13.5 dams were fixed in formalin (Thermo scientific) for at least two days before embedment in optimal cutting temperature compound. H&E staining was performed at the Histopathology Laboratory Core of UTMB.

### siRNA knockdown for Peli1

Cells were transfected with 37.5 pM control siRNA or Peli1 siRNA (Santa Cruz Biotechnology) per manufacturer’s instructions. Transfected cells were grown in RPMI medium containing 10% FBS. qPCR was used to confirm the efficiency of Peli1 siRNA knockdown. At 24 h post transfection, cells were infected with ZIKV. Supernatants and cells were harvested at 96 h pi to measure viral load and cytokine production.

### Cell death detection assay

Culture supernatants (10 μl) were transferred into a black 96-well plate (Costar). The adenylate kinase detection reagent (50 μl) (ToxiLight BioAssay, Lonza) was added to each well and the plate was incubated for 5 min at room temperature. Luminescence was measured in a microplate luminometer (Applied Biosystems). The results were expressed as relative luminescent unit (RLU).

### *In vivo* treatment with Smaducin-6

A129 mice were injected subcutaneously (s.c.) with 5 x 10^5^ PFU of ZIKV-PRV. At 2 h pi, 100 ug/kg of Smaducin-6 or Pal-Scram peptides [3232] (Sigma) were injected i.p., followed by three additional injections at 12 h intervals (a total of 16 mg/kg).

### Statistics

Survival curve comparisons were performed using Prism software (GraphPad) statistical analysis, which uses the log rank test. Values for viral burden, cytokine production, and fetus weight and size were presented as means ± SEM. P values of these experiments were calculated with a non-paired 2-tailed Student’s t test. Statistical significance was accepted at P < 0.05.

## Supporting information

S1 Fig Peli1 promotes ZIKV infection, mediates inflammatory responses, and exacerbates congenital diseases in pregnant mice. WT and *Peli1*^*-/-*^ mice were pretreated with MAR1-5A3 at E5.5 followed by infection with 1x10^4^ FFU ZIKV-FSS13025 (**A-D**) or ZIKV Dakar strain (**E**) one day later. The weight (**A**) and size (**B,** CRL x OF diameter) of 14–28 fetuses collected at E17.5 from non-infected and ZIKV-infected WT and *Peli1*^*-/-*^ dams. **C.** Placental diameter. ^##^ P < 0.01 compared to non-infected (NF) group. ** P < 0.01 or * P < 0.05 compared to WT group (Unpaired t test). **D-E.** Viral loads in fetal head tissues or fetal residues isolated from 7–9 fetuses or 11–12 fetuses at E17.5 (**D**) or E13.5 (**E**), respectively. ** P < 0.01 compared to WT group (Unpaired t test). **F-G**. Maternal blood cytokine levels were measured by qPCR. Data are presented as the fold increase compared to NF group and represent the means ± SEM of 5 samples. *P < 0.05 compared to WT group (Unpaired t test).
(TIF)

S2 Fig Peli1 expression in human first trimester placental trophoblasts following ZIKV infection. HTR8 cells were infected with ZIKV-FSS13025 (MOI = 10). At indicated times pi, cells were fixed with 4% paraformaldehyde. **A.** Immunodetection of Peli1 (green), ZIKV antigen (red), and Dapi (blue) at 4 h and 24 h pi. **B.** Immunodetection of ER (green), Peli1 (red), and Dapi (blue) at 4 h pi. **C.** Immunodetection of Peli1 (green), dsRNA (red), and Dapi (blue) at 4 h pi.
(TIF)

S3 Fig Smaducin-6 treatment in first trimester placental trophoblasts during ZIKV infection. **A**. HTR8 cells were infected with ZIKV-FSS13025 and treated with 50 and 200 nM Smaducin-6 or control peptides at 1 h pi. Cell death was determined at day 4 by the activity of adenylate kinase in culture supernatants. Data are presented as means ± SEM, n = 3–8. **B-C**. HTR8 cells were infected at MOI of 1 with ZIKV-PRV and treated with 100 nM Smaducin-6 or control peptides at 1 h pi. **B.** Viral load was measured at day 4 pi by qPCR assay. Data are presented as the means ± SEM of 6 samples pooled from 2 independent experiments. ** P < 0.01 compared to control group (Unpaired t test). **C.** Cytokine levels were measured at day 4 by qPCR. Data are presented as fold increase compared to mock-infected and are the representative of 2 independent experiments. n = 3. ** P < 0.01 or *P < 0.05 compared to control group.
(TIF)

S4 Fig The effects of Smaducin-6 on ZIKV life cycle. **A-B.** The effects of Smaducin-6 treatment on ZIKV attachment and entry. HTR8 cells were infected with ZIKV-FSS13025 (MOI = 10) and treated with Smaducin-6 or control peptide (100 nM) for 1 h at 4°C, washed, and collected to measure intracellular viral RNA by qPCR in the attachment assay (**A**). For virus entry (**B**), cells were subsequently resuspended in medium and incubated at 37°C for 4 h. Cells were washed to determine intracellular viral RNAs, n = 6. **C-D.** The effects of Smaducin-6 treatment on ZIKV infectivity. HTR8 cells were infected at MOI of 1 with viruses passaged once in control and Smaducin-6 treated HTR8 cells. Cytokine production (**C**) and viral load (**D**) were measured by qPCR at day 4 pi. Cytokine levels are presented as the fold increase compared to NF group. Data shown are representative of two similar experiments and are presented as means ± SEM, n = 4.
(TIF)

S5 Fig The effects of Smaducin-6 treatment *in vitro* and *in vivo* following ZIKV infection. **A-B.** A129 mice were infected with 5 x10^5^ PFU ZIKV-PRV, followed by injection with control or Smaducin-6 peptide 2 h pi and three additional treatments with a 12 h interval, n = 4 mice per group. At day 3 pi, viral loads in blood (**A**) and spleen tissues (**B**) were measured by qPCR. **C-E.** Smaducin-6 treatment in AB6 macrophages during ZIKV infection. BM-macrophages were infected at MOI of 0.1 with ZIKV-FSS13025 and treated with Smaducin-6 or control peptides at 1 h pi. **C.** Viral load was measured at day 4 pi by QPCR. **D-E.** Cytokine levels are presented as the fold increase compared to NF group. Data are presented as means ± SEM, n = 4. **F-G**. WT and *Peli1*^*-/-*^ macrophages were blocked with (MAR1-5A3, 125ug/ ml) followed by ZIKV-FSS13025 infection (MOI = 2) and treated with Smaducin-6 or control peptides at 1 h pi. Viral load (**F)** and IL-12 RNA levels (**G**) were measured at day 4 pi by qPCR. No significance (ns) indicates *P* > 0.05 compared to control group. **H.** The effects of Smaducin-6 treatment on maternal viral infection in pregnant A129 mice. A129 mice were infected with 5 x10^5^ PFU ZIKV-PRV on E6.5, followed by injection with control or Smaducin-6 peptide 2 h pi and three additional treatments every 12 h. At E13.5, viral loads in maternal blood and spleens were measured by qPCR. Data are presented as the means ± SEM of 4–5 samples per group. ** P < 0.01 compared to control group (Unpaired t test).
(TIF)
