# Supplementary figures and images for: Peli1 signaling blockade attenuates congenital zika syndrome
Source: PLoS Pathog. 2020 Jun 16;16(6):e1008538. doi: 10.1371/journal.ppat.1008538 (PMC7297310; doi:10.1371/journal.ppat.1008538)

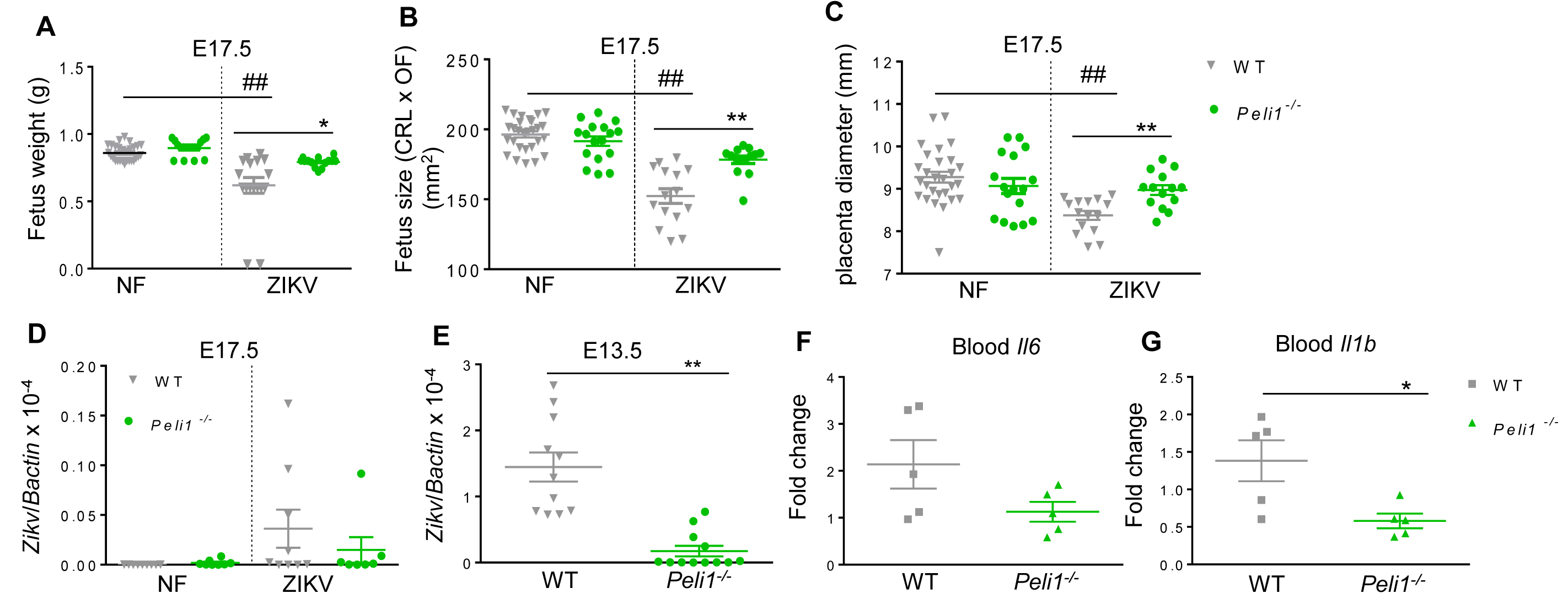

Supplement: S1 Fig — WT and Peli1-/- mice were pretreated with MAR1-5A3 at E5.5 followed by infection with 1x10^4^ FFU ZIKV-FSS13025 (A-D) or ZIKV Dakar strain (E) one day later. The weight (A) and size (B, CRL x OF diameter) of 14–28 fetuses collected at E17.5 from non-infected and ZIKV-infected WT and Peli1-/- dams. C. Placental diameter. ## P < 0.01 compared to non-infected (NF) group. ** P < 0.01 or * P < 0.05 compared to WT group (Unpaired t test). D-E. Viral loads in fetal head tissues or fetal residues isolated from 7–9 fetuses or 11–12 fetuses at E17.5 (D) or E13.5 (E), respectively. ** P < 0.01 compared to WT group (Unpaired t test). F-G. Maternal blood cytokine levels were measured by qPCR. Data are presented as the fold increase compared to NF group and represent the means ± SEM of 5 samples. *P < 0.05 compared to WT group (Unpaired t test). (TIF) [file ppat.1008538.s001.tif]

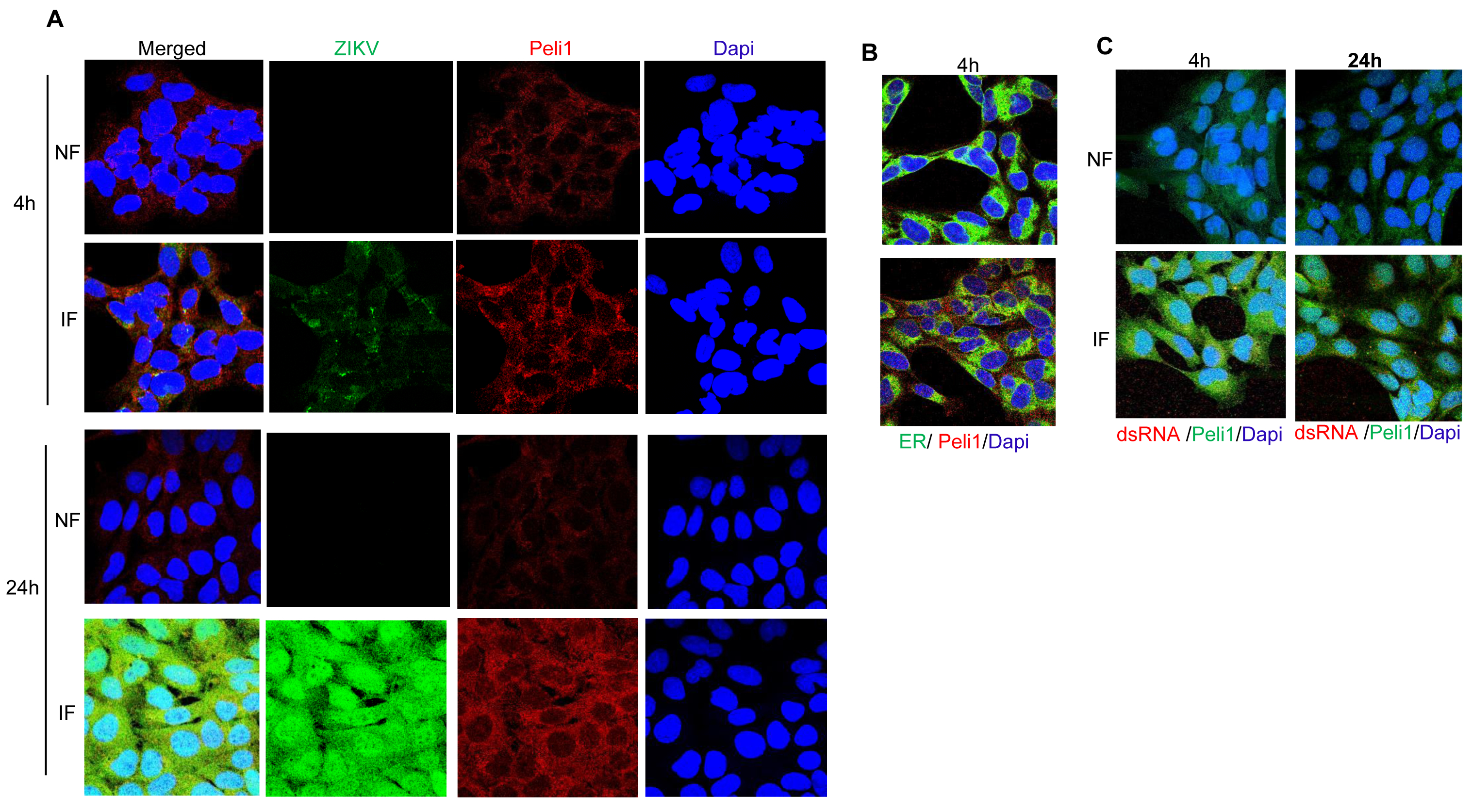

Supplement: S2 Fig — HTR8 cells were infected with ZIKV-FSS13025 (MOI = 10). At indicated times pi, cells were fixed with 4% paraformaldehyde. A. Immunodetection of Peli1 (green), ZIKV antigen (red), and Dapi (blue) at 4 h and 24 h pi. B. Immunodetection of ER (green), Peli1 (red), and Dapi (blue) at 4 h pi. C. Immunodetection of Peli1 (green), dsRNA (red), and Dapi (blue) at 4 h pi. (TIF) [file ppat.1008538.s002.tif]

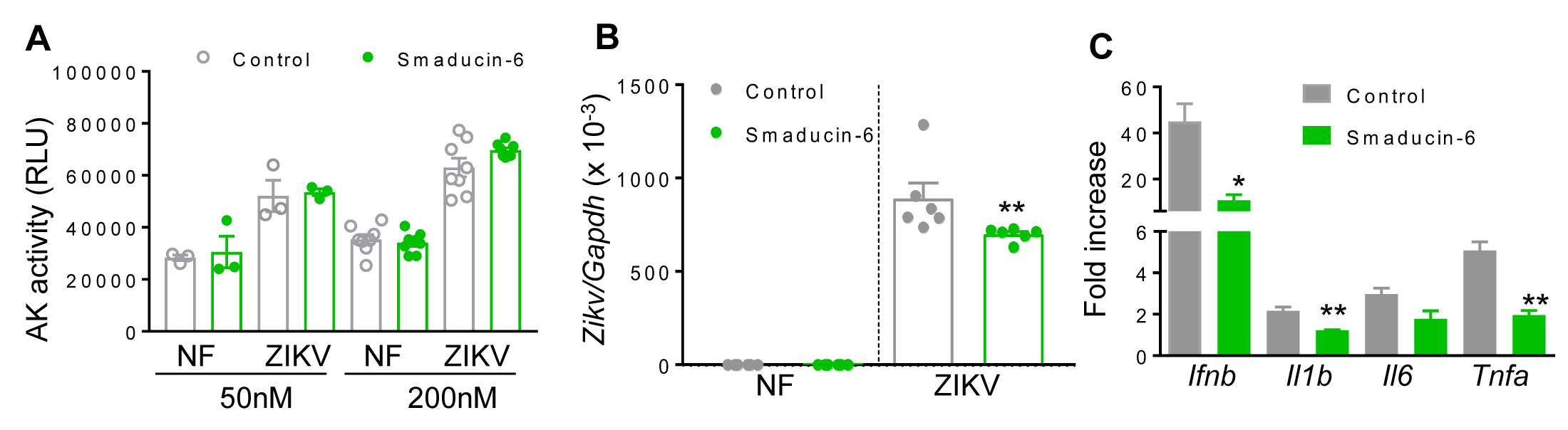

Supplement: S3 Fig — A. HTR8 cells were infected with ZIKV-FSS13025 and treated with 50 and 200 nM Smaducin-6 or control peptides at 1 h pi. Cell death was determined at day 4 by the activity of adenylate kinase in culture supernatants. Data are presented as means ± SEM, n = 3–8. B-C. HTR8 cells were infected at MOI of 1 with ZIKV-PRV and treated with 100 nM Smaducin-6 or control peptides at 1 h pi. B. Viral load was measured at day 4 pi by qPCR assay. Data are presented as the means ± SEM of 6 samples pooled from 2 independent experiments. ** P < 0.01 compared to control group (Unpaired t test). C. Cytokine levels were measured at day 4 by qPCR. Data are presented as fold increase compared to mock-infected and are the representative of 2 independent experiments. n = 3. ** P < 0.01 or *P < 0.05 compared to control group. (TIF) [file ppat.1008538.s003.tif]

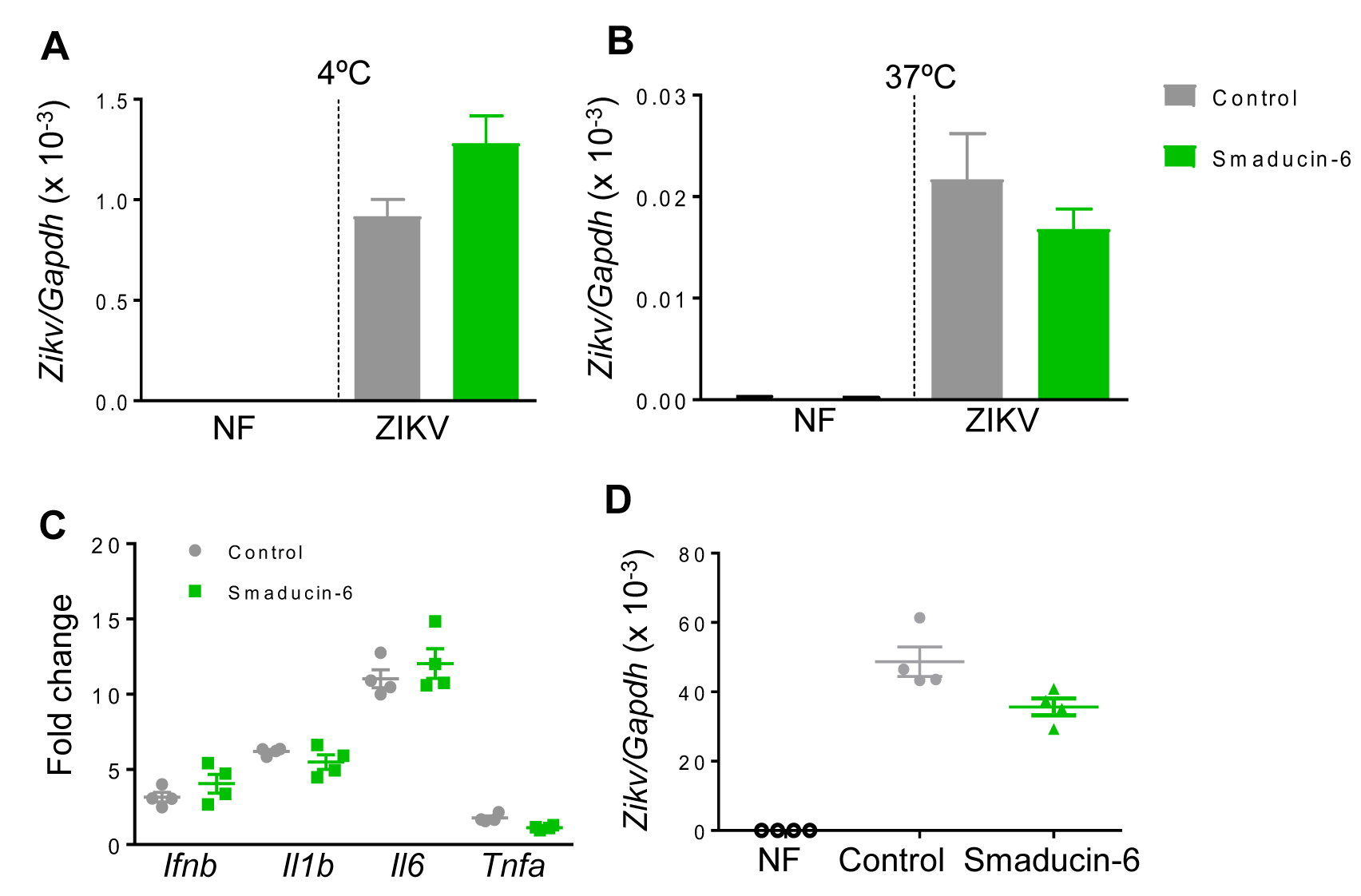

Supplement: S4 Fig — A-B. The effects of Smaducin-6 treatment on ZIKV attachment and entry. HTR8 cells were infected with ZIKV-FSS13025 (MOI = 10) and treated with Smaducin-6 or control peptide (100 nM) for 1 h at 4°C, washed, and collected to measure intracellular viral RNA by qPCR in the attachment assay (A). For virus entry (B), cells were subsequently resuspended in medium and incubated at 37°C for 4 h. Cells were washed to determine intracellular viral RNAs, n = 6. C-D. The effects of Smaducin-6 treatment on ZIKV infectivity. HTR8 cells were infected at MOI of 1 with viruses passaged once in control and Smaducin-6 treated HTR8 cells. Cytokine production (C) and viral load (D) were measured by qPCR at day 4 pi. Cytokine levels are presented as the fold increase compared to NF group. Data shown are representative of two similar experiments and are presented as means ± SEM, n = 4. (TIF) [file ppat.1008538.s004.tif]

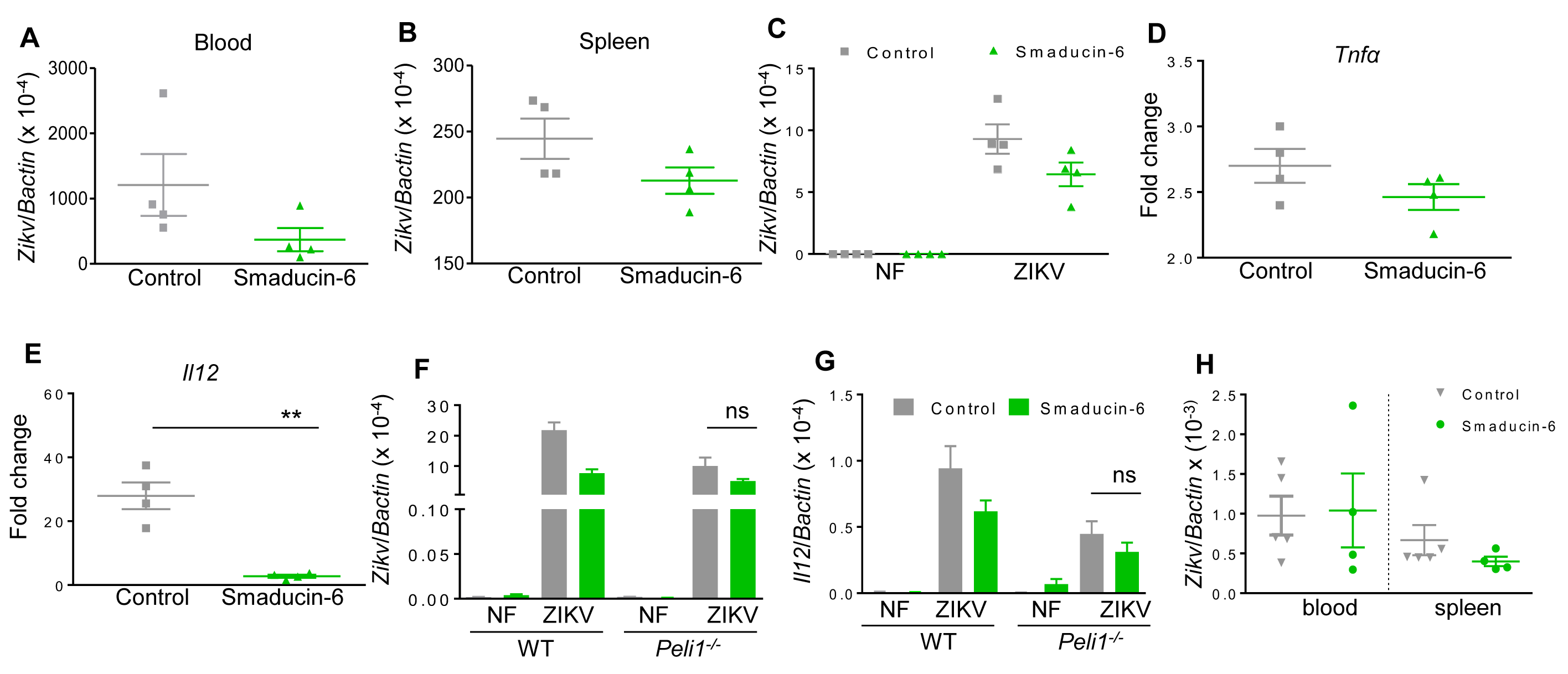

Supplement: S5 Fig — A-B. A129 mice were infected with 5 x10^5^ PFU ZIKV-PRV, followed by injection with control or Smaducin-6 peptide 2 h pi and three additional treatments with a 12 h interval, n = 4 mice per group. At day 3 pi, viral loads in blood (A) and spleen tissues (B) were measured by qPCR. C-E. Smaducin-6 treatment in AB6 macrophages during ZIKV infection. BM-macrophages were infected at MOI of 0.1 with ZIKV-FSS13025 and treated with Smaducin-6 or control peptides at 1 h pi. C. Viral load was measured at day 4 pi by QPCR. D-E. Cytokine levels are presented as the fold increase compared to NF group. Data are presented as means ± SEM, n = 4. F-G. WT and Peli1-/- macrophages were blocked with (MAR1-5A3, 125ug/ ml) followed by ZIKV-FSS13025 infection (MOI = 2) and treated with Smaducin-6 or control peptides at 1 h pi. Viral load (F) and IL-12 RNA levels (G) were measured at day 4 pi by qPCR. No significance (ns) indicates P > 0.05 compared to control group. H. The effects of Smaducin-6 treatment on maternal viral infection in pregnant A129 mice. A129 mice were infected with 5 x10^5^ PFU ZIKV-PRV on E6.5, followed by injection with control or Smaducin-6 peptide 2 h pi and three additional treatments every 12 h. At E13.5, viral loads in maternal blood and spleens were measured by qPCR. Data are presented as the means ± SEM of 4–5 samples per group. ** P < 0.01 compared to control group (Unpaired t test). (TIF) [file ppat.1008538.s005.tif]
